# Intestinal injury and changes of the gut microbiota after ischemic stroke

**DOI:** 10.3389/fncel.2025.1557746

**Published:** 2025-04-17

**Authors:** Yang Shen, Jin Wang, Yina Li, Xianhui Kang, Lijuan Gu

**Affiliations:** ^1^Department of Anesthesiology, Renmin Hospital of Wuhan University, Wuhan, China; ^2^Central Laboratory, Renmin Hospital of Wuhan University, Wuhan, China; ^3^Department of Anesthesiology, The First Affiliated Hospital, College of Medicine, Zhejiang University, Hangzhou, China

**Keywords:** gut microbiota, intestinal injury, ischemic stroke, individualized therapy, stroke

## Abstract

Stroke is the second leading cause of death and the third leading cause of disability worldwide, with ischemic stroke (IS) accounting for the vast majority of cases. This paper reviews the latest research on intestinal damage, changes in the gut microbiota, and related therapeutic strategies after IS. Following IS, the integrity of the intestinal mucosal barrier is compromised, leading to increased intestinal permeability. The gut microbiota can translocate to other organs, triggering systemic immune responses that inhibit recovery after IS. Moreover, the composition and proportion of the gut microbiota change after IS. The number of beneficial bacteria decreases, whereas the number of harmful bacteria increases. The production of beneficial metabolites, such as short-chain fatty acids (SCFAs), is reduced, and the levels of harmful metabolites, such as trimethylamine N-oxide (TMAO), increase. Antibiotics after IS not only help prevent infection but also have neuroprotective effects. Although poststroke reperfusion therapy can effectively restore cerebral blood flow, it may also cause intestinal mucosal damage and gastrointestinal dysfunction. Nutritional support after IS can alter the gut microbiota structure and promote neurological recovery. Therefore, individualized treatment for IS patients is crucial. In summary, IS affects not only the brain but the entire body system, especially the gut. Intestinal damage and dysbiosis are critical in IS occurrence, development, and prognosis. By protecting the intestinal mucosa and modulating the structure of the gut microbiota, intestinal damage and related infections can be reduced, improving patient prognosis. Future research is needed to explore therapeutic methods targeting the gut microbiota, providing more comprehensive and effective treatment strategies for IS patients.

## 1 Introduction

Stroke is the second leading cause of death worldwide, and the burden of disability after stroke is substantial. In 2020, stroke accounted for 6.6 million deaths, making it the second leading cause of death after neonatal disorders in children and ischemic heart disease in adults. It also represents the third leading cause of disability, resulting in 143 million disability-adjusted life years (Vos et al., 2020; Feigin et al., 2024). Stroke can be classified into hemorrhagic stroke or ischemic stroke (IS) based on its underlying pathological processes. The diagnosis of stroke is usually done using computed tomography (CT) of the brain, which is typically the first imaging study used to detect stroke and can distinguish between hemorrhagic and ischemic stroke, and the majority are IS (62.4%) (Walter, 2022). Currently, neurorestorative treatments are being tried to improve the neurological functions and daily life quality in patients with stroke (Huang H. et al., 2021; Huang et al., 2024; Zheng et al., 2023), but the limitation of stroke therapy is still existing due to the incompletely clear mechanisms. IS triggers two cascades of immune responses: in brain tissue, it manifests as inflammation and ischemic tissue damage; peripherally, it results in the migration of peripheral immune cells into the central nervous system, causing peripheral immune suppression and increasing the likelihood of systemic infection. Peripheral immune suppression after IS may increase intestinal susceptibility to infection and inflammation.

Patients suffering from IS often experience gastrointestinal dysfunction, which is typically characterized by impaired intestinal motility, changes in the intestinal microbiota composition, and inflammation (Tuz et al., 2022). Intestinal inflammation after IS is attributed mainly to the overactivation of immune cells and disruption of the intestinal epithelial barrier. This intestinal barrier breakdown leads to the translocation of the gut-dwelling microbiota, which spreads to other organs, exacerbating systemic immune responses and inhibiting recovery following IS. Interestingly, some metabolites produced by the gut microbiota after ischemic stroke can reduce brain ischemia–reperfusion injury by inhibiting poststroke inflammatory responses and promoting neurological function recovery (Hu et al., 2022). Maintaining the gut microbiota and repairing intestinal damage after IS may positively affect IS treatment (Pluta et al., 2021).

In this review, we briefly discuss the changes related to intestinal injury and the gut microbiota after IS and share our perspectives on personalized treatment for IS patients.

## 2 Intestinal injury after ischemic stroke

### 2.1 Changes in the intestinal mucosa after ischemic stroke

Typically, the gastrointestinal mucosa is exposed to various antigens at the luminal surface while maintaining close contact with the immune system through subepithelial lymphoid tissue (Kagnoff, 2014; Onyiah and Colgan, 2016). Thus, the monolayer epithelium of the gastrointestinal mucosa serves as a critical barrier that isolates luminal substances from the underlying mucosal immune system. A study by Liu et al. revealed significant morphological changes in the small intestine of mice after middle cerebral artery occlusion (MCAO), with obvious damage to the intestinal villous epithelium that worsened over time. At 6 h after IS, necrotic sloughing of epithelial cells was observed in the apical portions of a few villi; at 12 h after IS, almost all the villi lacked apical epithelium; at 24 h after IS, necrotic sloughing of epithelial cells and dissociation of the epithelial layer were observed in all the villi (Liu et al., 2017). Xu et al. reported similar findings, noting damage to the intestinal mucosa, including the villi, under both light and electron microscopes. Vacuolar degeneration of organelles, widening of cell-to-cell junctions, and the presence of apoptotic cells were also observed. Elevated levels of D-lactic acid, a byproduct of bacterial metabolism, may indicate bacterial translocation to the gut. Intestinal fatty acid-binding protein (IFABP) is an enzyme found in the cytoplasm of intestinal epithelial cells, and its presence in serum may indicate intestinal mucosal damage (Xu et al., 2012). A study by Carlos et al. revealed that the median serum concentrations of IFABP and D-lactic acid in patients with acute ischemic stroke (AIS) were significantly greater than those in controls. These findings suggest that the intestinal mucosal barrier is impaired in AIS patients (Camara-Lemarroy et al., 2021).

Disruption of the intestinal mucosal barrier often leads to changes in intestinal permeability, which is considered a quantifiable indicator of intestinal mucosal barrier function (Bischoff, 2011; Brandtzaeg, 2011). A study by Joshua et al. suggests intestinal permeability increases after IS and correlates with IS severity (Crapser et al., 2016). Research conducted by Chen et al. revealed that intestinal permeability increases after IS, and herbal formulas such as Puerariae radix (PLR) and Chuanqiong (CXR) decoctions effectively repair damage to the intestinal mucosal barrier caused by IS. Interestingly, their study also suggested that stroke-induced intestinal mucosal barrier dysfunction does not spontaneously improve but rather worsens over time (Chen et al., 2019). Research by Chen et al. further indicated that modulation of the gut microbiota through oral nonabsorbable antibiotics and fecal microbiota transplantation could alleviate the increased intestinal permeability caused by IS, thereby contributing to restoring the intestinal mucosal barrier (Chen et al., 2019). Zhang et al. suggested that atorvastatin promotes intestinal mucosal barrier function restoration by altering the gut microbiota composition. Therefore, intestinal mucosa changes could serve as a target for systemic treatment after IS (Zhang et al., 2021).

### 2.2 Alterations in intestinal mucosal immunity after ischemic stroke

The intestinal mucosal immune system consists of three distinct mucosal lymphoid structures: the epithelium, the lamina propria, and Peyer’s patches (PPs) (Richards et al., 2016; Zhang N. et al., 2022). The epithelial cells are coated with a layer of mucus rich in antimicrobial peptides, which directly influence the gut microbiota and activate intestinal adaptive immunity (Ayabe et al., 2004). The lamina propria, located beneath the epithelial cells of the intestine, is composed of T cells and B cells. Immune cells in the lamina propria can rapidly respond to abnormal gut signals, initiating inflammatory and anti-inflammatory responses. CD4^+^ T cells in the lamina propria secrete IL-17 and IL-22, which contribute to intestinal inflammation regulation (Lui et al., 2015). PPs are sites where intestinal B cells mature and produce secretory IgA, which is distributed throughout the small intestine. Humans typically have 100 ~ 200 PPs, whereas mice have 6–12 patches (Reboldi and Cyster, 2016). Secretory IgA is a crucial component of intestinal mucosal immunity (Bergqvist et al., 2010; Bemark et al., 2012).

Research by Ge et al. revealed that at 7 days after IS, the number of CD4^+^ T cells in the ileum significantly decreased, whereas the number and frequency of IL-17A-secreting *γ*δT cells significantly increased. Similar immune phenotypes were observed 14 days after IS. These findings suggest that IS suppresses the immune response of ileal CD4^+^ T cells, affecting both the ileal microbiota and epithelial function (Ge et al., 2022). A study by Liu et al. revealed a significant increase in T lymphocytes in PPs after IS, whereas the number of B lymphocytes and intraepithelial lymphocytes did not change significantly (Liu et al., 2017). Research by Vikramjeet et al. revealed that the abundance of proinflammatory IL-17^+^ Th17 and IFN-γ^+^ Th1 cells in PPs increases more than fourfold after a large cerebral infarction. In addition, many T cells migrate from the gut to the peri-infarct tissue after IS, exacerbating the damage caused by IS (Singh et al., 2016). Benakis et al. suggested that T cells migrated from the gut to the meninges after IS and that the accumulation of IL-17^+^ γδT cells in the meninges was associated with increased infarct size (Benakis et al., 2016).

**Figure 1:**
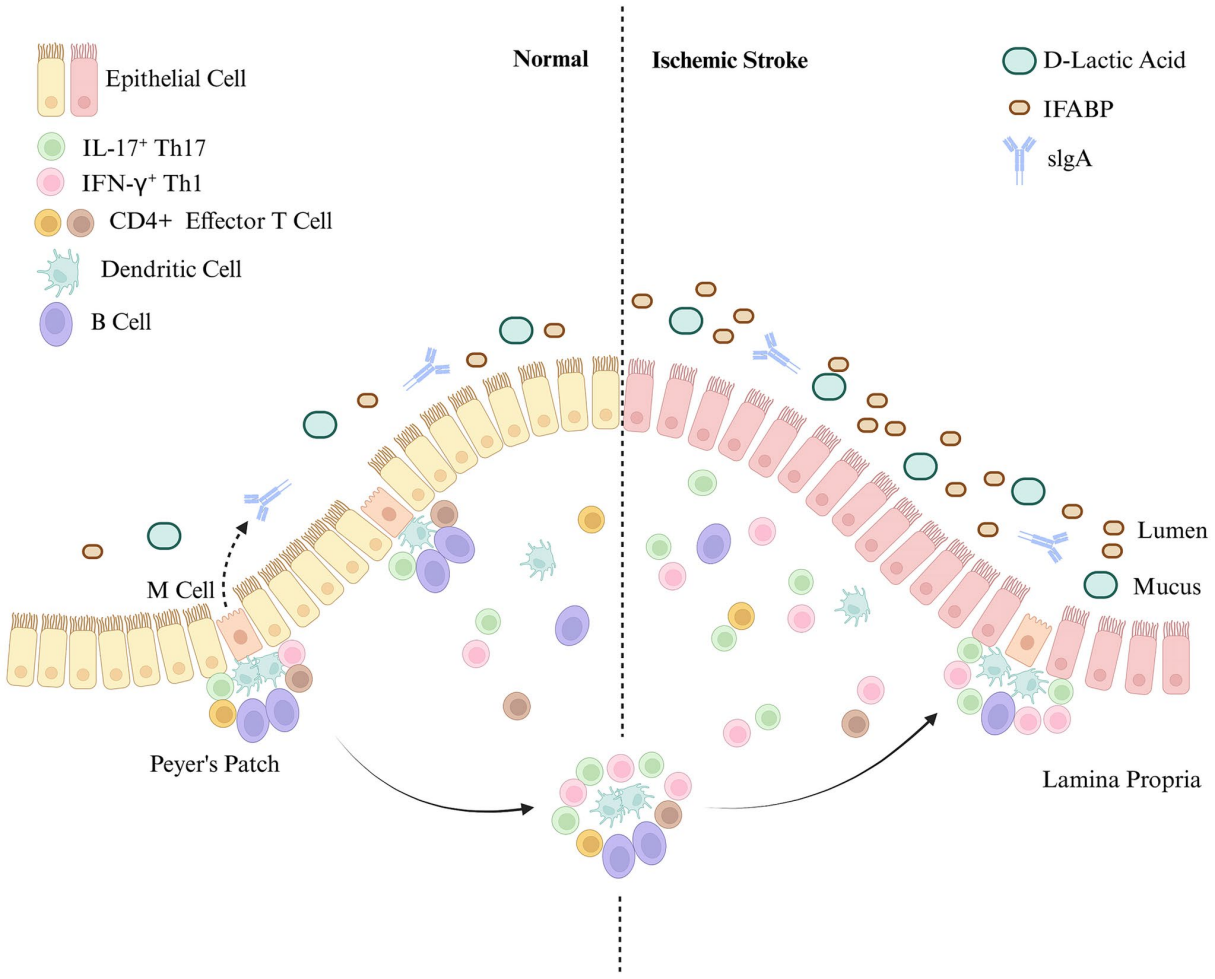
Intestinal mucosal immunity after ischemic stroke. The intestinal mucosal immune system consists of three distinct mucosal lymphoid structures: the epithelium, the lamina propria, and Peyer’s patches (PPs). The intestinal mucosal barrier is damaged after IS. Intestinal mucosal immune function is significantly enhanced, with immune cells in the intestinal mucosa (including PPs) primarily consisting of T cells. In contrast, the increase in B cells and endothelial cells is not obvious.

Changes in gut mucosal immunity after IS are closely related to the pathophysiological mechanisms and prognosis of IS. Targeting these changes may be critical for improving gastrointestinal complications after IS (see Figure 1).

## 3 Changes in the gut microbiota after ischemic stroke

### 3.1 Diversity of the gut microbiota

The gut microbiota consists primarily of bacteria, fungi, and viruses, with viruses making up approximately 98% of the microbial community. The total symbiotic microbial community in the gut weighs approximately 0.2–1.0 kg (Wang et al., 2023). The genera Bacteroidia and Bacillota make up approximately 90% of the gut microbiota. Previous research has shown that changes in the quantity and proportions of the gut microbiota can lead to various pathological and physiological conditions. The balance of the gut microbiota plays a critical role in maintaining the metabolism and immune balance of the body. The amount and proportions of the gut microbiota vary in different parts of the gastrointestinal tract.

The stomach was once thought to be sterile due to gastric acid sterilization and bile reflux, but *Helicobacter pylori (H. pylori)* has been detected in the stomach. The microbiota in the stomach primarily originates from the oral cavity. In healthy individuals, the stomach may harbor bacteria such as *Lactobacillus* spp., *Streptococcus* spp., *Veillonella* spp., *Rothia* spp., and *Haemophilus* spp. (Nardone and Compare, 2015). The small intestine is divided into three parts: the duodenum, jejunum, and ileum. Due to sufficient oxygen availability, bacterial density and diversity are limited in the duodenum, with the microbiota primarily consisting of Actinobacteria and Bacillota (El Aidy et al., 2015). The jejunum supports colonization by facultative anaerobic bacteria due to relatively limited oxygen levels. The microbiota in this region consists primarily of *Lactobacillus* spp., *Enterococcus* spp., and *Streptococcus* spp. (El Aidy et al., 2015). The microbiota of the ileum, which is closer to the proximal end and resembles the jejunum, is dominated by aerobic bacteria. In contrast, the portion near the ileocecal valve, resembling the colon, is predominantly populated by anaerobic bacteria (Adak and Khan, 2019). Due to the slow movement of food and the anaerobic environment in the large intestine, the microbiota is predominantly composed of anaerobic bacteria (Eckburg et al., 2005). The bacterial density is highest in the colon, where the microbiota is dominated by the genera Bacteroidia and Bacillota (Mariat et al., 2009). In addition to bacteria, fungi account for 0.1% of the total number of the gut microbiota (Zhang F. et al., 2022) and they play a crucial role in human intestinal homeostasis and the pathogenesis of diseases (Sender et al., 2016; Nash et al., 2017). Fungi colonize the gut before birth. In infants, the gut fungal community is initially dominated by the genera *Candida* spp. and *Malassezia* spp. during the first month (Willis et al., 2019). Subsequently, Malassezia spp. gradually declines to undetectable levels by the age of 5 months (Fujimura et al., 2016). Diet plays a significant role in shaping the gut microbiota (Schei et al., 2017; Zhang F. et al., 2022). Subsequently, the gut fungal community undergoes further changes and matures into an adult-like fungal community, characterized by a substantial increase in the diversity of the gut fungal community, with a compositional predominance of Ascomycota, Basidiomycota, and Zygomycota (Hallen-Adams et al., 2015; Nash et al., 2017). The types, amounts, and proportions of the gut microbiota can vary under different pathological and physiological conditions and may serve as therapeutic targets for corresponding diseases.

### 3.2 Changes in the gut microbiota after ischemic stroke

Many studies have suggested the existence of a bidirectional communication network between the brain and the gut, known as the gut–brain axis. The gut can influence cognition and behavior in the brain through the gut–brain axis, whereas the brain can transmit mental stress to the gastrointestinal tract through the same axis. The gut microbiota can influence neurodevelopment, cognition, and behavior through this pathway. After IS, the integrity of the intestinal mucosal barrier is compromised, leading to changes in the gut microbiota. Research by Jeon et al. revealed that in a porcine model of IS, the diversity and evenness of the gut microbiota decreased on the first day after IS. In addition, the diversity of the gut microbiota 3 days after IS was negatively correlated with the lesion volume (Jeon et al., 2020). A study by Chen et al. suggested that the impact of IS on the gut microbiota composition varies depending on the severity of IS (Gu et al., 2021). A study by Lou et al. revealed that changes in the gut microbiota are observed in AIS patients compared with healthy controls, characterized by an imbalance in the ratio of short-chain fatty acid-producing bacteria to pathogenic bacteria (Lou et al., 2024). According to the research of Tan et al., patients with AIS have a gut microbiota lacking short-chain fatty acid-producing bacteria, especially in severe cases (Tan et al., 2021). According to a study by Sun et al., IS patients with a poor prognosis at 3 months exhibit baseline dysbiosis in the gut microbiota. This dysbiosis is characterized by an increase in the abundance of pathogenic bacteria and a decrease in the abundance of bacteria that produce short-chain fatty acids (Sun et al., 2022). Interestingly, Li et al. reported that certain bacteria that produce short-chain fatty acids are more abundant in patients with IS than in healthy individuals (Li et al., 2019).

### 3.3 Alterations in the relative abundance of beneficial and harmful intestinal bacteria after ischemic stroke

After IS, the composition of the gut microbiota changes, leading to alterations in the abundance of both beneficial and harmful bacteria. According to a study by Han et al., the abundance of harmful bacteria in the gut increases, whereas that of beneficial bacteria decreases in IS patients (Han et al., 2024). Wang et al. reported that in patients with IS, the abundances of the families Lactobacillaceae, Enterococcaceae, Streptococcaceae, and Enterobacteriaceae are positively correlated with risk factors and negatively correlated with preventive factors (Zhang N. et al., 2022). A study by Kazuo et al. revealed that the abundance of *Lactobacillus* spp. in the intestine is significantly greater after IS than in control patients. In addition, the variation in *Lactobacillus* spp. infection rates was positively correlated with serum Interleukin-6 levels, suggesting a close association between the variation in Lactobacillus abundance and intestinal inflammation (Yamashiro et al., 2017). Lei et al. reported that the most abundant bacteria in the gut after IS were *Pseudomonas* spp., *Acinetobacter* spp., and *Akkermansia* spp. (Xiang et al., 2020). In conclusion, the gut microbiota composition after IS is characterized by an increased abundance of harmful bacteria and decreased beneficial bacteria.

### 3.4 Impact of ischemic stroke on gut microbiota metabolites

**Figure 2:**
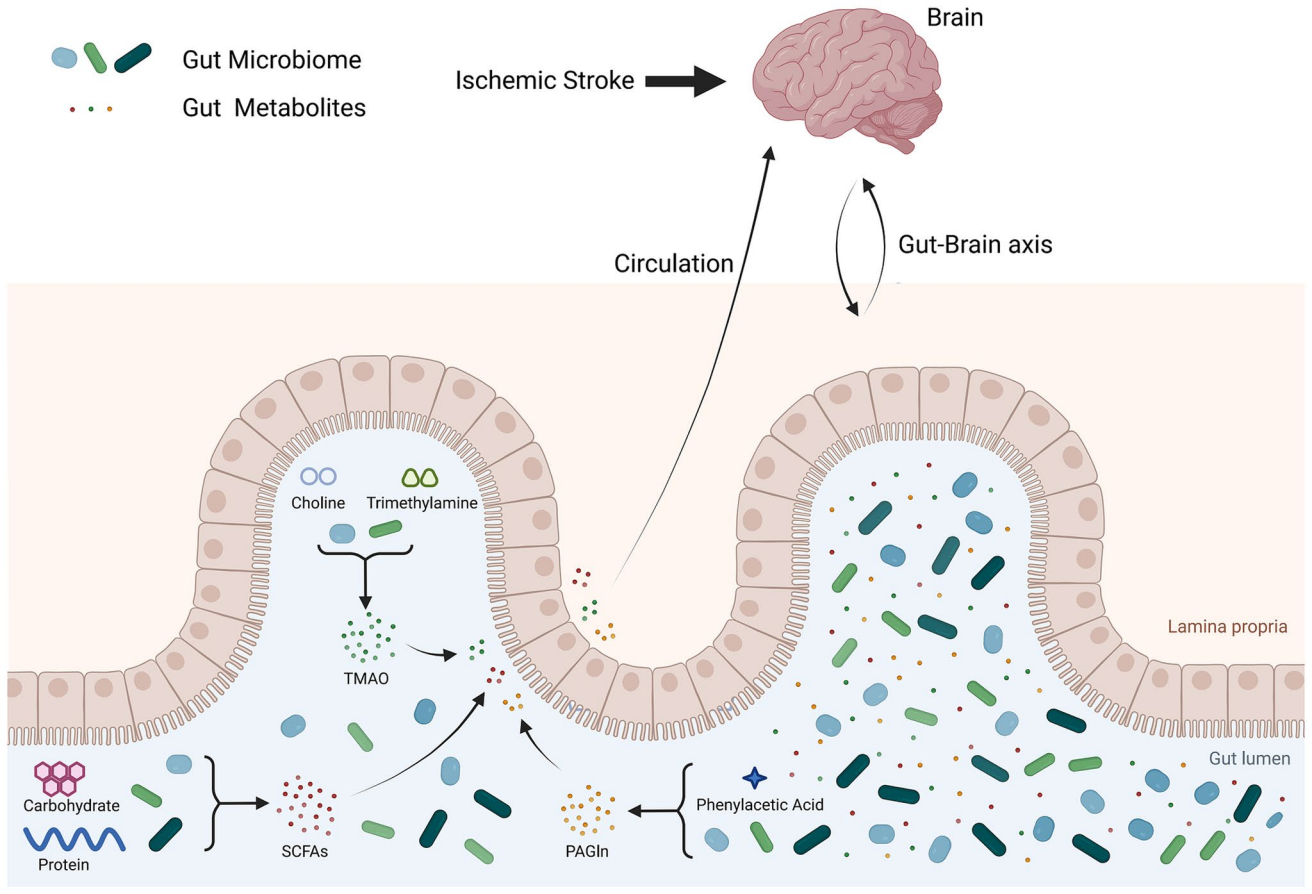
Gut microbiota metabolites after ischemic stroke. After IS, the intestinal mucosa is damaged, leading to an imbalance in the intestinal flora and increased levels of metabolites produced by the gut microbiota. These gut microbiota metabolites enter the blood circulation through the damaged intestinal mucosa and affect the brain.

The gut microbiota produces various metabolic products, including short-chain fatty acids (SCFAs), trimethylamine N-oxide (TMAO), lipopolysaccharides (LPS), and phenylacetylglutamine (PAGln). These products have a significant effect on the cardiovascular system. According to the research of Zhao et al., stroke-associated differentially abundant metabolites were more evident in feces than in plasma and urine (Zhao et al., 2022). SCFAs are produced primarily by beneficial bacteria in the intestines and are then transported into the body. According to Chen et al., patients with IS have decreased production of SCFAs by their gut bacteria (Chen et al., 2021). According to Kazuo et al., IS was associated with acetic and pentanoic acid levels. The acetic acid concentration was negatively correlated with glycated hemoglobin and low-density lipoprotein levels, whereas the pentanoic acid concentration was positively correlated with C-reactive protein and white blood cell count (Yamashiro et al., 2017). Nicholas et al. reported that the concentration of plasma SCFAs is not correlated with IS severity at onset. However, higher levels of SCFAs are linked to increased levels of inflammatory markers, slower recovery between admission and discharge, and poorer functional status at discharge (Henry et al., 2022). According to Song et al., high levels of SCFAs in the bloodstream were linked to severe poststroke complications (Chou et al., 2023b). TMAO is a metabolic waste product produced by the gut microbiota. It is considered a metabolite associated with IS and other cardiovascular diseases (Xu et al., 2021). Research by Sun et al. revealed a positive correlation between plasma TMAO levels and IS (Sun et al., 2021). According to a study by Wang et al., elevated levels of plasma TMAO upon admission may indicate adverse clinical outcomes in patients with AIS (Zhai et al., 2019). According to Wang et al., the level of TMAO decreased over time after the onset of IS. Additionally, early elevated levels of TMAO following IS indicate adverse IS outcomes (Tan et al., 2020). Research by Song et al. suggested that the level of TMAO is not related to the severity of IS or functional prognosis (Chou et al., 2023b). PAGln is a metabolite produced by the intestinal microbiota that can activate platelets and increase the risk of thrombosis, potentially leading to cardiovascular and cerebrovascular events. According to research by Yu et al., patients with AIS have elevated plasma PAGln levels correlated with white matter hyperintensity burden (Yu et al., 2021). Therefore, the metabolism of the intestinal microbiota is closely associated with the occurrence and prognosis of IS. Measuring the levels of metabolites related to the intestinal microbiota can predict the occurrence and severity of IS (see Figure 2).

## 4 Influencing factors and preventive strategies for ischemic stroke

### 4.1 The impact of antibiotic use on the gut microbiota after ischemic stroke

The use of antibiotics in patients with AIS not only serves to prevent infection but also offers neuroprotective effects (Van De Beek et al., 2009). For example, minocycline, with its various anti-inflammatory properties, can reduce microglial cell activation, inhibit apoptosis, and has shown favorable outcomes in experimental IS studies (Yong et al., 2004). In rodent models of IS, ceftriaxone has been shown to reduce mortality and ameliorate neurological deficits (Thöne-Reineke et al., 2008). However, post-stroke use of antibiotics can affect the gut microbiota. According to research by Benakis et al., these changes in the gut microbiota after IS can result in increased numbers of regulatory T cells and a reduced number of IL-17^+^ γδT cells by altering dendritic cell activity, ultimately alleviating ischemic brain injury in mice (Benakis et al., 2016). Benakis et al. reported that mice treated with antibiotics during the acute phase of IS presented a reduced cerebral infarct volume (Benakis et al., 2020). Chen et al. demonstrated that oral administration of nonabsorbable antibiotics post-stroke could reduce neuronal damage and cerebral infarct volume by altering the gut microbial composition (Chen et al., 2019). Therefore, antibiotics can be considered adjunctive therapies for IS.

### 4.2 Reperfusion therapy and intestinal injury after ischemic stroke

Post-stroke reperfusion therapy, which includes intravenous thrombolysis, intra-arterial thrombolysis, mechanical thrombectomy, and stent placement, is the most effective treatment for patients with AIS (Powers et al., 2019). Additionally, Song et al. reported a correlation between gut microbiota changes and reperfusion therapy outcomes in these patients (Chou et al., 2023a). Isobutyric acid, an SCFA associated with gut microbiota metabolism, enters the bloodstream due to damage to the intestinal mucosal barrier after IS. According to Yang et al., elevated plasma isobutyric acid levels are associated with the prognosis of patients undergoing reperfusion therapy for AIS (Chou et al., 2023b). Reperfusion therapy may cause local-to-systemic pathological damage, such as ischemia–reperfusion injury, oxidative stress, and calcium overload, in patients with AIS. This can result in secondary damage to the intestines. To date, there has been limited research on this topic.

### 4.3 Nutritional support and microbiota modulation after ischemic stroke

Severe IS patients often present with gastrointestinal dysfunction and require either enteral or parenteral nutritional support. According to Tian et al., the gut microbiota of IS patients who receive enteral nutrition significantly changes, possibly related to patient prognosis (Tian et al., 2022). Modulating the gut microbiota through probiotic use can also impact IS prognosis. Han et al. demonstrated that probiotic administration may influence IS prognosis by improving the intestinal environment. Bifico, a probiotic, was orally administered for 28 days, reducing cerebral infarct volume in IS model mice and promoting neurological recovery. This effect may be due to the modulation of brain tissue immune function and inflammatory response mechanisms (Han et al., 2024). Mao et al. reported that administering Lactobacillus to IS model mice promoted long-term recovery (Huang J.-T. et al., 2021). Lian et al. suggested that probiotic intervention is effective for IS because it improves the gut microbiota (Lian et al., 2023). Inducing the gut microbiota toward a normal state after IS benefits IS recovery and prognosis.

### 4.4 Necessity of individualized treatment after ischemic stroke

AIS patients are typically middle-aged or elderly, and men have a higher incidence than women. Age is a significant risk factor for IS. Park et al. reported that transferring the gut microbiota from adult female mice that had undergone ovariectomy and estrogen depletion to middle-aged female mice that had undergone ovariectomy and estrogen depletion significantly reduced the cerebral infarct volume and accelerated behavioral recovery (Park et al., 2020). Gender is also a risk factor for IS. Giorgio et al. reported that men who have suffered from IS exhibit higher levels of intestinal permeability and IL-17 than women (Guido et al., 2022). However, it is still unclear how sex-related differences in the gut microbiota impact post-stroke functional deficits.

Long-term treatment for IS typically includes antiplatelet and lipid-lowering therapy. Chen et al. reported that long-term oral administration of aspirin and atorvastatin alters the gut microbiota, affecting their preventive effects against IS (Chen et al., 2023). Zhang et al. demonstrated that atorvastatin administration is associated with restoring the gut microbiota, improving intestinal barrier function, and modulating intestinal immune function in IS model mice (Zhang et al., 2021). After IS, adjunctive rehabilitation therapies, such as electrotherapy, are available. Zhang et al. reported that electroacupuncture significantly improved neurological function and intestinal injury in IS model rats, restored the gut microbiota, and regulated intestinal immunity (Zhang et al., 2023). Zhao et al. demonstrated that administering electroacupuncture-treated rat feces to IS model mice without electroacupuncture treatment could better alleviate brain injury (Li et al., 2022). Herbal medicine is another crucial adjunctive treatment for IS. Wang et al. demonstrated that Zhilong Huoxue Huayu capsules, a traditional Chinese medicine effective for treating IS, can reduce cerebral infarct volume, improve neurological function, and alleviate intestinal injury in IS model rats (Wang et al., 2022). Guo et al. suggested that charcoal soup may alleviate IS by modulating the abundance of metabolites, including endotoxins and TMAO, in the gut microbiota and the gut microbiota, which exacerbates sterile inflammation and platelet aggregation (Guo et al., 2021). Xian et al. demonstrated that brain-nourishing soup is an effective traditional Chinese medicine formula for treating IS. Compared with monotherapy, combination therapy with acupuncture is superior, effectively reducing infarct volume, alleviating oxidative stress and inflammatory responses, and regulating intestinal microbiota dysbiosis (Xian et al., 2022).

Recurrent stroke tends to worsen existing symptoms, requiring stricter standardized treatment. Yang et al. demonstrated that, compared with mice that have undergone a single stroke, those that have experienced recurrent stroke had significantly lower survival rates and increased cerebral infarct volumes. Both groups experienced significant changes in the gut microbiota (Yang et al., 2024).

Stroke treatment should be personalized, with a rational treatment plan based on each patient’s circumstances (see Table 1).

**Table 1:** Literature review related to influencing factors and preventive strategies for ischemic stroke.

Title	Journal	Author	Year
Antibiotics			
Preventive antibiotics for infections in acute stroke: a systematic review and meta-analysis	Archives of Neurology	van de Beek D, Wijdicks EFM, etc.	2009
The promise of minocycline in neurology	Lancet Neurol	Yong VW, Wells J, etc.	2004
The beta-lactam antibiotic, ceftriaxone, dramatically improves survival, increases glutamate uptake and induces neurotrophins in stroke	Journal of Hypertension	Namsolleck P, Schmerbach K, etc.	2008
Commensal microbiota affects ischemic stroke outcome by regulating intestinal γδT cells	Nature Medicine	Benakis C Brea D, etc.	2016
Distinct commensal bacterial signature in the gut is associated with acute and long-term protection from ischemic stroke	Stroke	Benakis C, Poon C, etc.	2020
Transplantation of fecal microbiota rich in short chain fatty acids and butyric acid treat cerebral ischemic stroke by regulating gut microbiota	Pharmacological Research	Chen R, Xu Y, etc.	2019
Reperfusion therapy			
The prognostic biomarkers of plasma trimethylamine N-oxide and short-chain fatty acids for recanalization therapy in acute ischemic stroke.	International Journal of Molecular Sciences	Chou P-S, Yang IH, etc.	2023
Predicting adverse recanalization therapy outcomes in acute ischemic stroke patients using characteristic gut microbiota	Microorganisms	Chou P-S, Hung W-C, etc.	2023
Nutritional support			
Effect of enteral nutrition on the intestinal microbiome and risk of death in ischemic stroke patients	Journal of Parenteral and Enteral Nutrition	Tian X, Xia G, etc.	2022
Bifico ameliorates neurological deficits after ischemic stroke in mice: transcriptome profiling	In vivo (Athens, Greece)	Han Y, Xu H, etc.	2024
Calorie restriction conferred improvement effect on long-term rehabilitation of ischemic stroke via gut microbiota	Pharmacological Research	Huang J-T, Mao Y-Q, etc.	2021
Gut microbiota-derived melatonin from Puerariae Lobatae radix-resistant starch supplementation attenuates ischemic stroke injury via a positive microbial co-occurrence pattern	Pharmacological Research	Lian Z, Xu Y, etc.	2023
Individualized treatment			
Reproductive senescence and ischemic stroke remodel the gut microbiome and modulate the effects of estrogen treatment in female rats	Translational Stroke research	Park MJ, Pilla R, etc.	2023
Sex and age dimorphism of the gut-brain axis in ischemic stroke: a systematic review of preliminary studies	Brain Research	Guido G, Crivellaro E, etc.	2022
Effects of long-term regular oral aspirin combined with atorvastatin to prevent ischemic stroke on human gut microbiota	European Journal of Pharmacology	Chen G, Wang Z, etc.	2023
Atorvastatin alleviates microglia-mediated neuroinflammation via modulating the microbial composition and the intestinal barrier function in ischemic stroke mice	Free Radical Biology & Medicine	Zhang P, Zhang X, etc.	2021
Electroacupuncture and human iPSC-derived small extracellular vesicles regulate the gut microbiota in ischemic stroke via the brain-gut axis.	Frontiers in Immunology	Zhang Q, Deng P, etc.	2023
Gut flora mediates the rapid tolerance of electroacupuncture on ischemic stroke by activating melatonin receptor through regulating indole-3-propionic acid	The American Journal of Chinese Medicine	Li S, Zhao X, etc.	2022
Zhilong Huoxue Tongyu capsules’ effects on ischemic stroke: an assessment using fecal 16S rRNA gene sequencing and untargeted serum metabolomics	Frontiers in Pharmacology	Wang R, Liu M, etc.	2022
Gut microbiota-related effects of Tanhuo decoction in acute ischemic stroke	Oxidative Medicine and Cellular Longevity	Guo Q, Jiang X, etc.	2021
Integrated 16S rRNA gene sequencing and LC/MS-based metabolomics ascertained synergistic influences of the combination of acupuncture and NaoMaiTong on ischemic stroke	Journal of Ethnopharmacology	Xian M, Shen L, etc.	2022
Systemic characterization of the gut microbiota profile after single mild ischemic stroke and recurrent stroke in mice	Biomedicines	Yang D, Sun P, etc.	2024

## 5 Conclusion

This article discusses intestinal damage, gut microbiota changes after IS, and treatments targeting these specific issues. Post-stroke gastrointestinal dysfunction is caused by intestinal injury and changes in the composition and translocation of the gut microbiota. Assessing gastrointestinal function is essential for IS patients. Systemic treatment for IS is crucial for patient prognosis and rehabilitation. Targeted interventions can be implemented post-stroke to protect the intestinal mucosa and restore normal gut microbiota structure, thereby reducing intestinal damage and associated infections. Individualized treatment should be provided based on the specific needs of each patient. Improving gastrointestinal function in ischemic stroke patients is a crucial adjunctive therapy. Healthcare providers should pay significant attention to and actively manage post-stroke gastrointestinal dysfunction.

Ischemic stroke is a systemic disease that originates in the brain but manifests throughout the body. Undoubtedly, treatment targeting the infarcted area is crucial. However, systemic therapy is also highly valuable, and treatment targeting the gut microbiota is one of the most essential aspects. We believe that there will be more research and therapeutic prospects in this area in the future.
